# Subsequent treatments beyond progression on osimertinib in EGFR-mutated NSCLC and leptomeningeal metastases

**DOI:** 10.1186/s12916-022-02387-0

**Published:** 2022-05-30

**Authors:** Mei-Mei Zheng, Yang-Si Li, Hai-Yan Tu, Hao Sun, Kai Yin, Ben-Yuan Jiang, Jin-Ji Yang, Xu-Chao Zhang, Qing Zhou, Chong-Rui Xu, Zhen Wang, Hua-Jun Chen, De-Xiang Zhou, Yi-Long Wu

**Affiliations:** 1 School of Medicine, South China University of Technology, Guangzhou, 510006 China; 2 Guangdong Lung Cancer Institute, Guangdong Provincial People’s Hospital, Guangdong Academy of Medical Sciences, Guangzhou, 510080 China; 3 Guangdong Provincial Key Laboratory of Translational Medicine in Lung Cancer, Guangdong Provincial People’s Hospital, Guangdong Academy of Medical Sciences, Guangzhou, 510080 China; 4 Department of Neurosurgery, Guangdong Provincial People’s Hospital, Guangdong Academy of Medical Sciences, Guangzhou, 510080 China

**Keywords:** Cerebrospinal fluid, Leptomeningeal metastases, Osimertinib

## Abstract

**Background:**

Despite the reported efficacy of osimertinib, central nervous system (CNS) progression is still frequent in *EGFR*-mutated NSCLC. This study aimed to reveal site-specific resistant mechanisms to osimertinib and investigate subsequent treatments for leptomeningeal metastases (LM).

**Methods:**

*EGFR*-mutated NSCLC with LM who progressed on osimertinib were included. Molecular analysis of cerebrospinal fluid (CSF) at osimertinib progression was performed. Subsequent treatments of LM were collected and analyzed.

**Results:**

A total of 246 patients were identified. Only those with LM as a progression site on osimertinib were included (*n*=81). In 58 CSF-plasma pairs, more alterations were uniquely detected in CSF (77%) than in plasma (7%). These mechanisms led to 22 patients receiving matched targeted therapy. Among them, 16 (72.7%) had a clinical response. The median overall survival was 7.2 months. For non-matched therapy (*n*=59), the osimertinib combination had a longer median overall survival than the regimen switch in CNS-only progression (15.3 vs. 7 months, *p*=0.03). Finally, serial monitoring by CSF revealed the potential evolution of LM.

**Conclusions:**

Private resistant mechanisms in CSF might match osimertinib-resistant LM for targeted therapy. Besides, continuing osimertinib with intensification strategy might prolong survival, especially for those with CNS-only progression. Prospective  exploration is needed.

**Supplementary Information:**

The online version contains supplementary material available at 10.1186/s12916-022-02387-0.

## Background

Leptomeningeal metastasis (LM) is a complication of deeper concerns due to its increasing incidence rate, limited treatment options, and dismal survival outcomes in patients with *EGFR*-mutated non-small cell lung cancer (NSCLC) [1]. One major obstacle is the exclusion of patients with LM in most clinical trials [2]. Even though osimertinib has been proved to be effective for patients with *EGFR*-mutated LM, treatment after progression on osimertinib is still limited [3–5]. Another barrier is the genomic heterogeneity between primary tumor and LM [6, 7]. A recent, delicately designed trial has proved that central nervous system (CNS) metastasis patients from solid tumors can achieve intracranial benefit from targeted therapy informed by intracranial molecular testing [8]. But the inaccessibility to a biopsy of LM at progression hinders its further utility in patients with LM.

Therefore, it is important to identify molecular alterations and possible resistant mechanisms of LM in a less-invasive way and leverage them to specifically control LM. Our previous study has proved that cerebrospinal fluid (CSF) cell-free DNA (cfDNA) revealed unique genetic profiles of LM compared with tumor and plasma [7]. Furthermore, genotyping of CSF seemed to predict intracranial efficacy and show possible resistant mechanisms of osimertinib for LM patients [9]. These studies indicated that CSF was useful for molecular analysis of LM. However, it is unknown whether by detecting possible resistant mechanisms of osimertinib, a liquid biopsy of CSF can match these patients to subsequent targeted therapies and in predicting their therapeutic benefit of controlling LM.

In the absence of a resistant mechanism, biomarker-driven strategies are not feasible. Considering inadequate drug concentration in CSF is one reason for limiting response specific to CNS disease, dose intensification of osimertinib may be considered rather than a direct transition to an agnostic approach like chemotherapy as extracranial progressions usually do. Especially osimertinb has significantly greater CNS efficacy than earlier generation *EGFR*-tyrosine kinase inhibitors (TKIs) and should not be easily given up [3–5]. However, data on continuing osimertinib with intensifying strategy is lacking when patients are compliant with osimertinib standard dose and diagnosed with LM.

CNS progression was observed in only 6% of patients in the front-line osimertinib FLAURA trial, and the incidence of LM progression was less than that [10]. Here, we focused on patients with *EGFR*-mutated NSCLC who were heavily treated and then had LM as the progression site on osimertinib. Possible resistant mechanisms detected by paired CSF and plasma were shown, and serial CSF was also obtained. We presented subsequent treatment strategies including biomarker-directed therapies based on liquid biopsy of CSF and continuing osimertinib as non-matched treatments.

## Methods

### Patients

We screened patients with *EGFR*-mutated NSCLC who were diagnosed with LM in Guangdong Lung Cancer Institute from July 2016 to May 2021. The diagnosis of LM was assessed either by positive CSF cytology or typical leptomeningeal presentations on brain magnetic resonance imaging (pathological enhancement of the leptomeninges of the brain, cranial nerves, and spinal cord) and clinical neurological symptoms. Treatment histories were collected. Among those who were treated with osimertinib, reasons for osimertinib failure were further collected: (1) first diagnosis of LM during treatment with osimertinib, (2) progression of known LM, and (3) non-LM progression. Meeting the 1st and 2nd criteria allowed the inclusion in this study. Subsequent treatments after osimertinib failure were also collected and evaluated. The LM response assessment was based on modified Response Assessment in Neuro-Oncology (RANO)-LM radiological criteria [5]. Extracranial and brain parenchymal lesions control were assessed according to Response Evaluation Criteria in Solid Tumors (RECIST), version 1.1. Despite retrospective data collection, a clinical response which was defined as improvement or stabilization of neurologic symptoms and assessed by modified RANO-LM [5] was recorded in medical documents in our institute. So the clinical response was also included for analysis.

### Analysis of resistant mechanisms

CSF and paired plasma circulating tumor DNA (ctDNA) and tumor if available, was obtained and analyzed using the next-generation sequencing (NGS) at the time of osimertinib failure. The results were used to match patients with LM to receive targeted therapies if resistance mechanisms were detected. NGS was performed using targeted panels. DNA extraction, sequencing library preparation, and targeted capture enrichment were carried out following the methods as previously described [7, 11].

Included patients from our institute provided signed informed consent, and the study protocol was approved by the Research Ethics Committee of Guangdong Provincial People’s Hospital. The resistant mechanism landscape of 26 patients was reported and correlated to intracranial progression-free survival of osimertinib in our previous study [9].

### Statistical analysis

Overall survival (OS) was defined as the time from the start of subsequent treatment after osimertinib failure to death or last follow-up. The survival analysis was performed using the Kaplan-Meier method and compared with log-rank *p* values reported. *P* values were considered significant if less than 0.05. The χ^2^ test was used to analyze the patient characteristic of LM between matched and non-matched cohorts. SPSS version 26.0 (SPSS, Chicago, IL) was used for statistical analyses and GraphPad Prism version 8 (GraphPad Software Inc, San Diego, CA, USA) for making graphs.

## Results

### Patient characteristics

**Fig. 1:**
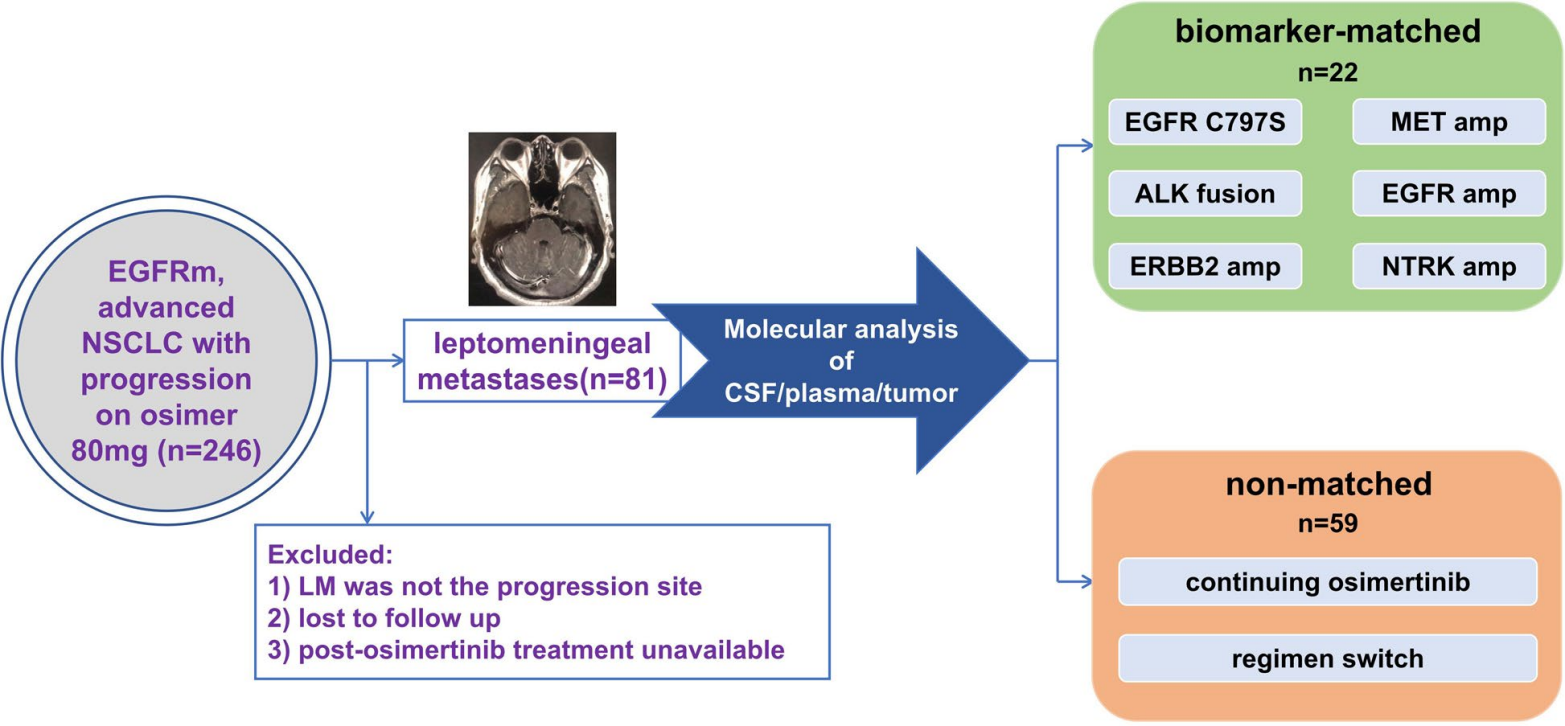
Treatment overview. Biomarker-matched targeted therapy was mainly based on molecular analysis of CSF. Osimer, osimertinib; CSF, cerebrospinal fluid; chemo, chemotherapy; bevac, bevacizumab

We studied a cohort of 246 patients with *EGFR*-mutated NSCLC who were diagnosed with LM and had a history of using osimertinib. Exclusion criteria were as follows: (1) LM was not the progression site, (2) lost to follow-up, and (3) subsequent treatments after progression on osimertinib were unavailable. Finally, a total of 81 patients with LM as the progression site on osimertinib were included for further analysis (Fig. 1).

Among them (median age 54, range 22–74 years), 37 patients were female and 61 never-smokers. All but 2 (adenosquamous) were histologically confirmed adenocarcinoma. At the initial diagnosis of NSCLC, 39 patients harbored *EGFR* exon 19 deletions, 34 had *EGFR* exon 21 *L858R* mutation, and 8 had uncommon *EGFR* mutations (*EGFR G719A*, *L747P*, *S768I*, and exon 20 insertions). Concurrent brain metastases (BM) were reported in 59 patients, and 25 received brain radiotherapy. What should be noticed was that the number of patients with an Eastern Cooperative Oncology Group performance status (ECOG-PS) score ≥2 was 37 (45.7%). The patients were heavily pre-treated with a median of 3 prior systemic therapies (range 1–7). Forty-nine (60.5%) of them progressed on known LM, and the remaining 32 (39.5%) had their first LM diagnosis (Table 1).

**Table 1:** Patient characteristics

Patient characteristics	Total number *n*=81 (%)	Matched *n*=22 (%)	Non-matched *n*=59 (%)	*P* value (matched vs. non-matched)
Median age, years (range)	54 (22-74)	54 (37-74)	54 (22-73)	/
Sex				
Female	37 (45.7%)	11 (50%)	26 (44.1%)	0.6
Male	44 (54.3%)	11 (50%)	33 (55.95)	
Smoking status				
Never	61 (75.3%)	18 (81.8%)	43 (72.9%)	0.4
Former	20 (24.7%)	4 (18.2%)	16 (27.1%)	
Histology type				
Adenocarcinoma	79 (97.5%)	22 (100%)	57 (96.6%)	1.0
Adenosquamous	2 (2.5%)	0	2 (3.4%)	
*EGFR* mutation status				
19DEL	39 (48.1%)	13 (59.1%)	26 (44.1%)	0.2
21L858R	34 (42.0%)	9 (40.9%)	25 (42.4%)	
Others*	8 (9.9%)	0	8 (13.5%)	
Concurrent brain metastases				
Yes	59 (72.8%)	13 (59.1%)	46 (78%)	0.09
No	22 (27.2%)	9 (40.9%)	13 (22%)	
No. of extracranial metastases				
0–1	48 (59.3%)	15 (68.2%)	33 (55.9%)	0.3
≥2	33 (40.7%)	7 (31.8%)	26 (44.1%)	
ECOG-PS				
0–1	44 (54.3%)	11 (50%)	33 (55.9%)	0.6
≥2	37 (45.7%)	11 (50%)	26 (44.1%)	
No. of prior systemic therapies	3 (1-7)	2 (1-7)	2 (1-4)	0.4
Prior brain radiotherapy				
Yes	25 (30.9%)	6 (27.3%)	19 (32.2%)	0.7
No	56 (69.1%)	16 (72.7%)	40 (67.8%)	
LM status				
PD on pre-existing LM	49 (60.5%)	11 (50%)	38 (64.4%)	0.2
First diagnosis of LM	32 (39.5%)	11 (50%)	21 (35.6%)	
Progression mode on osimertinib				
CNS-only progression	67 (82.7%)	18 (81.8%)	49 (83.1%)	1.0
Systemic progression	14 (17.3%)	4 (18.2%)	10 (16.9%)	

*EGFR G719X, L747P, S768I, and exon 20 insertion

*ECOG-PS* Eastern Cooperative Oncology Group-performance status, *TKI* Tyrosine kinase inhibitor, *osimer* osimertinib, *LM* leptomeningeal metastases; *PD* progression

### CSF-private resistant mechanisms to osimertinib compared to plasma

**Fig. 2:**
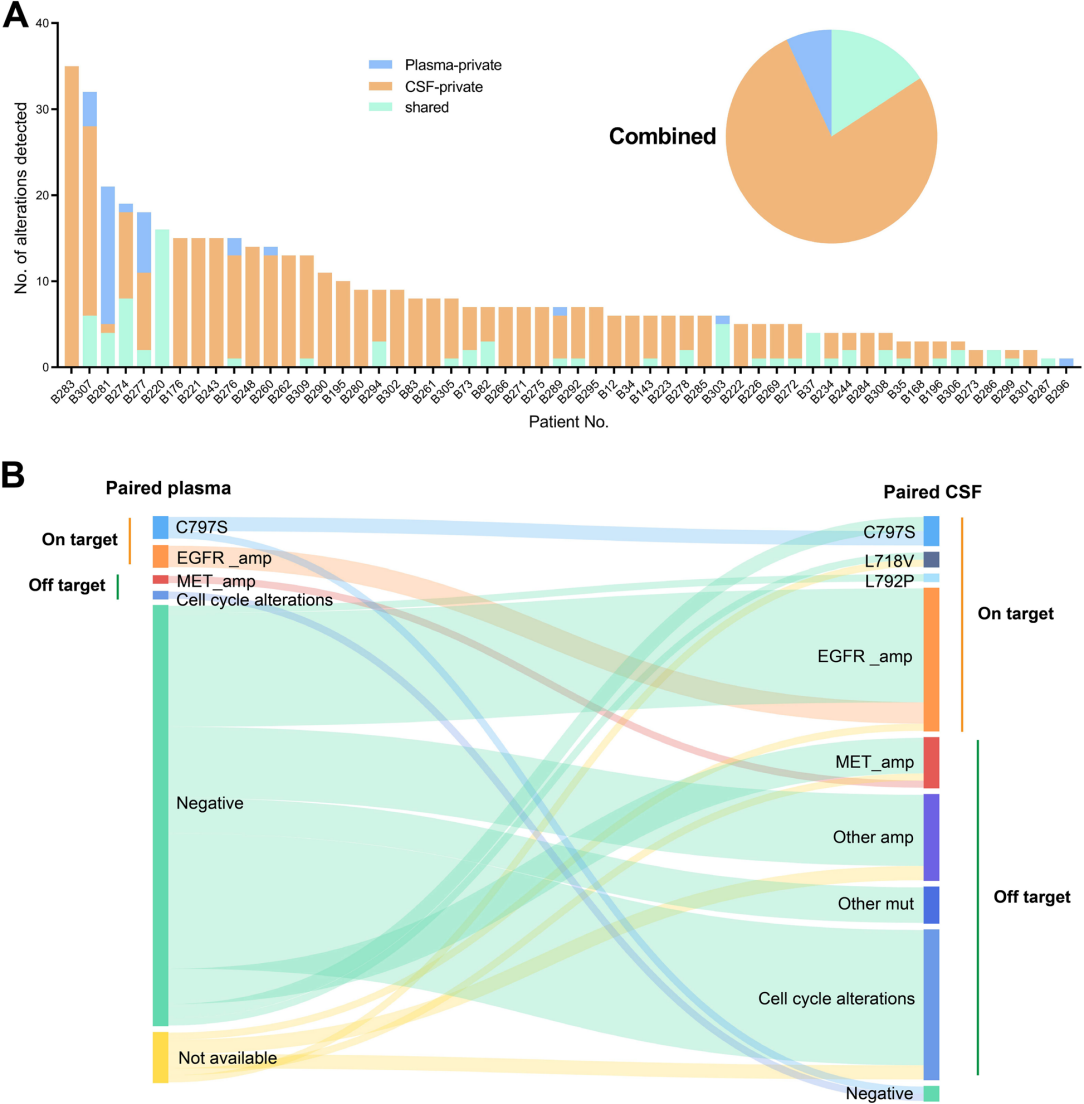
CSF cfDNA documents molecular characterization of EGFR-mutated NSCLC with LM. **A** Frequency of shared versus CSF-private or plasma-private alterations in paired CSF-plasma samples. **B** Discordance of on-target and off-target resistant mechanisms to osimertinib upon LM progression between paired CSF and plasma. CSF, cerebrospinal fluid; amp, amplification; mut, mutation

At the time of LM with acquired resistance to osimertinib, CSF cfDNA was available for 66 patients; paired plasma and tumor were obtained from 58 and 10 patients, respectively, for NGS. In the 58 paired CSF-plasma pairs which had at least one alteration detected, the percentage of shared genetic alterations varied across samples (range, 0–100%), with a median 10% shared mutation rate, which was considerably low. There was no shared mutation found in 48% of paired samples. More alterations were uniquely detected in CSF (77%) than in plasma (7%) (Fig. 2A). In the 10 paired CSF-tumor pairs, a median 47.2% shared mutation rate was seen. The percentages of unique alterations found in CSF and tumor were 38.5% and 16.5%, respectively. As a reference to previously reported mechanisms of resistance to osimertinib [12], discordance in the resistant mechanisms between paired CSF and plasma was present (Fig. 2B). For *EGFR* target-dependent resistance (on-target), *C797S* mutation was detected in 2 paired CSF-plasma and 2 additional CSF samples; *EGFR L718V* and *L792P* mutations were only found in CSF (*n*=3); *EGFR* gene amplification was detected in 20 CSF samples and only in 3 paired plasma. For *EGFR* target-independent mechanisms, *MET* amplification and cell cycle alterations were among the most frequently altered pathways both of which were mainly detected in CSF (*MET*, *n*=7; cell cycle, *n*=21), further verifying the superiority of CSF cfDNA for identifying unique genetic profiles of LM [7, 13]. As to paired CSF and tumor, a C797S mutation was detected both in CSF and tumor in 1 patient; 2 C797S mutations and 1 rare ALK fusion were detected only in tumor in 3 patients; Other than these, CSF-private and tumor-private mutations were still of unknown significance to disease progression.

### Matched targeted therapy based on CSF NGS results

**Fig. 3:**
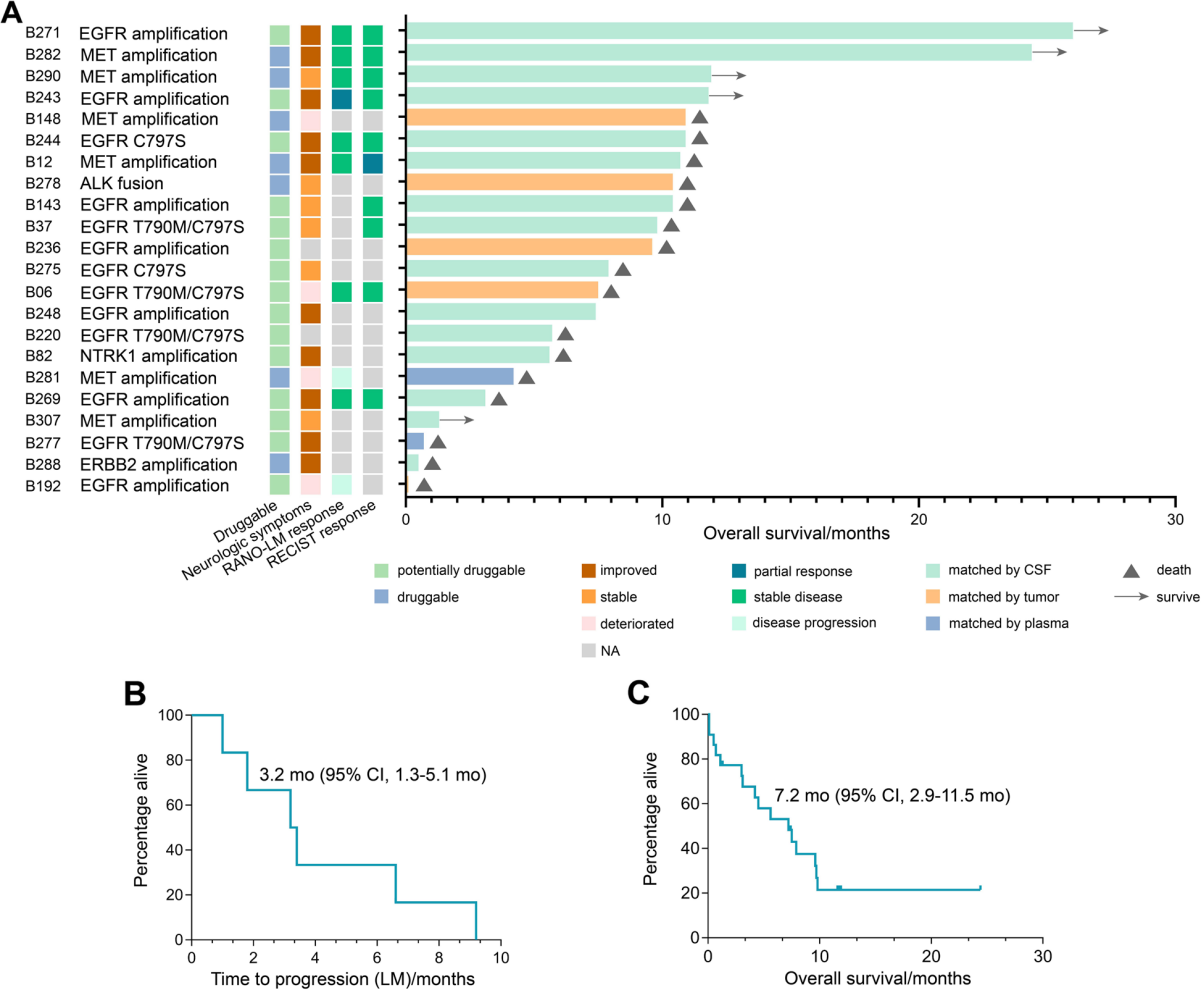
Matched targeted therapy for LM informed by CSF cfDNA. **A** Response and survival outcome of matched targeted therapy. **B** Time to LM progression from start of matched targeted therapy until LM progression for patients with intracranial benefit (partial response and stable disease). **C** Overall survival of matched targeted therapy; RANO-LM, Response Assessment in Neuro-Oncology-leptomeningeal metastases; RECIST, Response Evaluation Criteria in Solid Tumors; NA, not available; CSF, cerebrospinal fluid

After progression on osimertinib, NGS of CSF (*n*=15), plasma ctDNA (*n*=2), and extracranial tumor (*n*=5) identified known and possible resistant mechanisms that led to matched targeted therapy in 22 patients with LM (Fig. 3). Fifty percent of them had ECOG PS score ≥2, and patients have received a median of 2 prior systemic therapies (range 1–7). Conventional drugs approved by the Chinese Food and Drug Administration as well as therapies under investigation were used to target *C797S*, *MET* amplification, *ALK* fusion, *EGFR* amplification, etc., some of which were druggable and some of which were potentially druggable (Additional file 1: Table S1). The median time between genetic testing and matched targeted therapy was 21 days (range 1–468 days). Ten patients had clinical improvement, and 6 remained neurologically stable after the initiation of matched treatments (clinical response: 16/22, 72.7%). Among those with radiological evaluations available (*n*=10), 1 had a partial response (PR) and 7 had stable disease (SD) as their best LM response. One patient and 9 patients had PR and SD as their best extracranial responses, respectively (Fig. 3A). The median time from the start of matched targeted therapy until LM progression was 1.8 months (95% CI, 0.1–3.8 months). The median time from the start of matched targeted therapy until LM progression for patients with intracranial benefit (PR and SD) was 3.2 months (95% CI, 1.3–5.1 months) (Fig. 3B). The median time from the start of matched targeted therapy until death or last follow-up was 7.2 months (95% CI, 2.9–11.5 months, Fig. 3C). What should be taken special attention was that 1 patient received osimertinib combined with crizotinib to overcome *EGFR L858R* mutation and *MET* amplification detected in CSF for over 2 years and was still on treatment at last follow-up.

### Continuing osimertinib as non-matched therapy

**Fig. 4:**
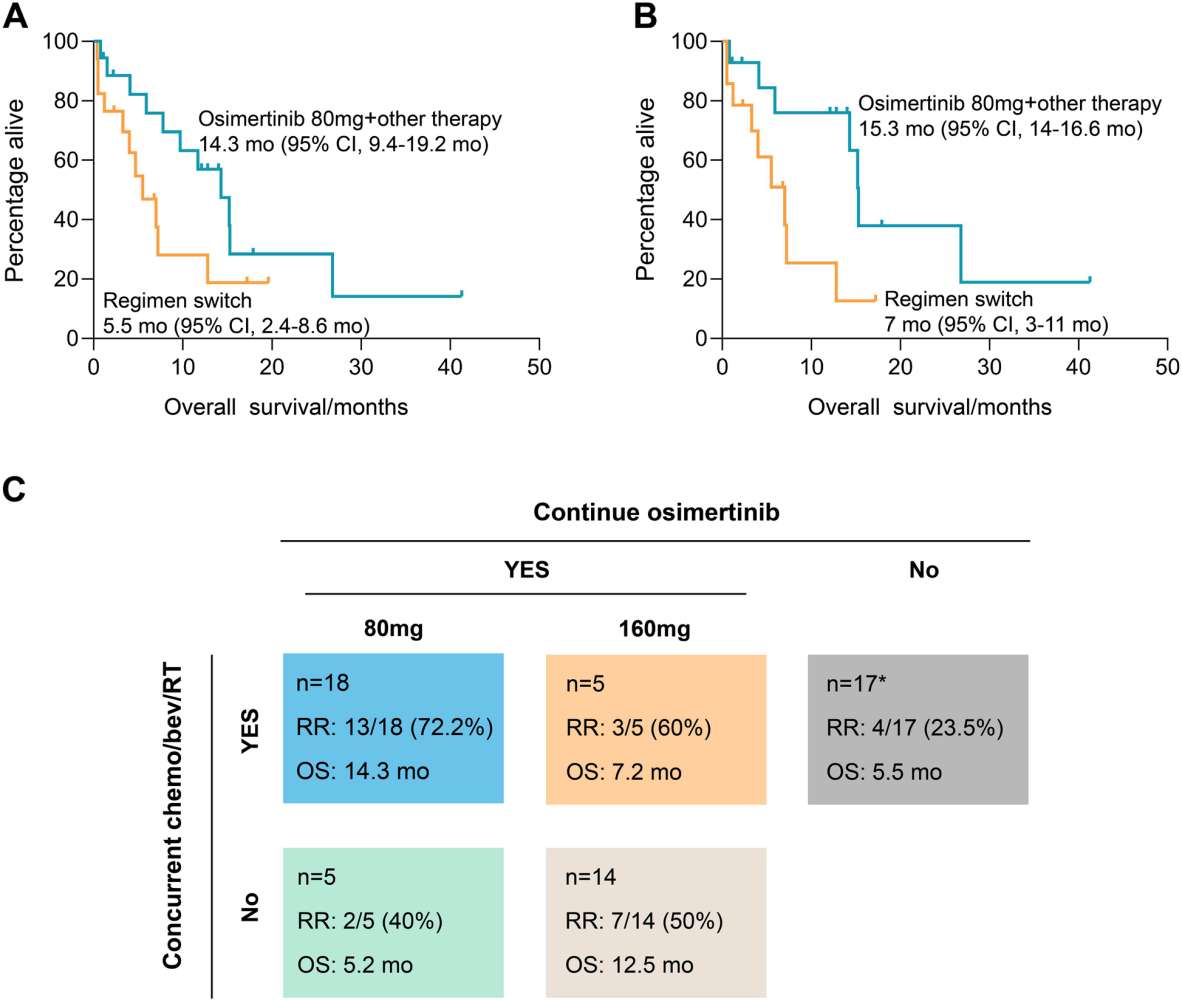
Non-matched therapy as post-osimertinib treatment for LM. **A** Comparison of overall survival between osimertinib 80 mg combination (chemotherapy/bevacizumab/radiotherapy) and regimen switch. **B** Comparison of overall survival between osimertinib 80 mg combination (chemotherapy/bevacizumab/radiotherapy) and regimen switch in those with central-nervous-system-only progression. **C** Clinical response and survival outcome of osimertinib 80 mg combination, dose escalation, osimertinib 160 mg combination, continuing osimertinib 80 mg, and regimen switch. mo, months; chemo, chemotherapy; bev, bevacizumab; RT, radiotherapy; RR: response rate (clinical); OS, overall survival

Regardless of known or potential resistant mechanisms detected at progression, 59 patients received non-matched therapy which was defined as continuing osimertinib or regimen switch without osimertinib. Median OS for the full cohort was 7.8 months (95%CI, 1.8–13.8 months), who had received a median of 2 prior systemic therapies (range 1–5), and 44.1% of whom had ECOG PS score ≥2 (Additional file 2: Fig. S1A). Eighteen patients received other therapy (chemotherapy/bevacizumab/radiotherapy, combination) with osimertinib 80 mg continuation, and 17 patients were treated with a switch regimen (Additional file 3: Table S2). The combination strategy had a trend towards longer survival than the regimen switch (14.3 months (95%CI, 9.4–19.2 months) vs. 5.5 months (95%CI, 2.4–8.6 months), *p*=0.09) (Fig. 4A). In patients with CNS-only progression, the osimertinib combination (*n*=14) achieved a significantly longer median OS than the regimen switch (*n*=14; 15.3 months vs. 7 months, *p*=0.03, no significant clinical confounder was found in this subset-of-subset analysis) (Fig. 4B), which was not seen in those with systemic progression (7.8 months vs. 4.7 months, *p*=0.8) (Additional file 2: Fig. S1B). Other regimens of continued osimertinib included another 14 patients who had osimertinib dose escalation, 5 continued osimertinib 80 mg alone, and 5 had osimertinib 160 mg combined with other therapy (dose escalation plus combination), median OS of which were 12.5 months, 5.2 months, and 7.2 months, respectively (Fig. 4C). Patients had 72.2% neurological response rate with osimertinib 80 mg combination (13/18), 50% with dose escalation (7/14), 60% with osimertinib 160 mg combination (3/5), 40% with continuing osimertinib 80 mg (2/5), and 23.5% with regimen switch (4/17) (Fig. 4C). For those who had radiological evaluations available (*n*=22), 9 patients had SD as their best LM response with osimertinib 80 mg combination; 4 patients had SD as their best LM response with dose escalation and 4 with regimen switch.

### Serial monitoring by CSF

**Fig. 5:**
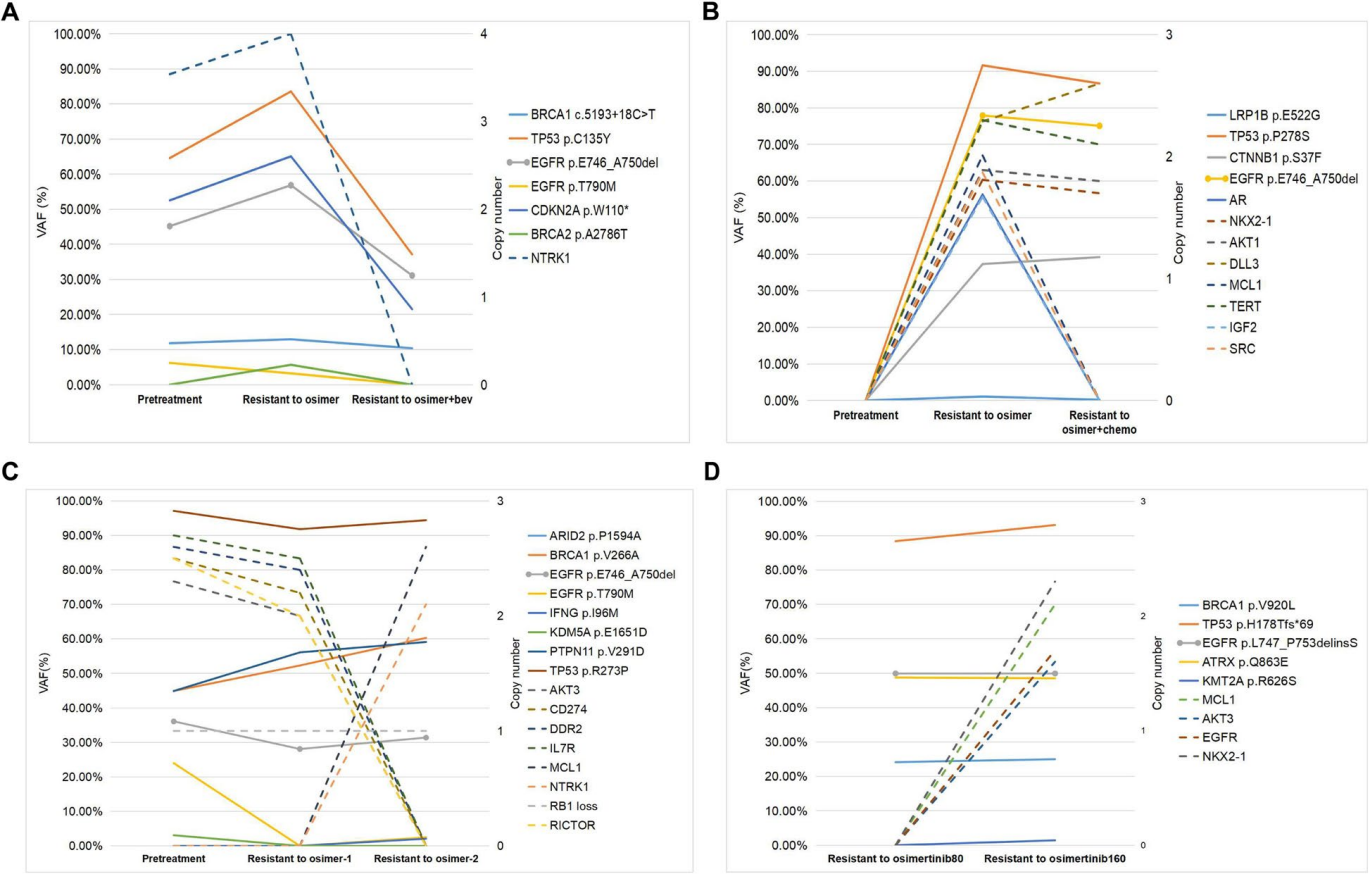
Serial monitoring by CSF for osimertinib treatment in patients with LM. **A** Patient B266: CSF cfDNA sequencing at baseline, disease progression during osimertinib 80 mg treatment, and second progression on osimertinib 80 m combined with bevacizumab. **B** Patient B176: CSF cfDNA sequencing at baseline, disease progression during osimertinib 80 mg treatment, and second progression on osimertinib 80 m combined with chemotherapy. **C** Patient B274: CSF cfDNA sequencing at baseline, disease progression during osimertinib 80 mg treatment, and second progression on continuing osimertinib 80 m; **D** Patient B222: CSF cfDNA sequencing at disease progression during osimertinib 80 mg treatment and second progression on osimertinib 160 mg. Full line indicated mutations annotated by the left vertical axis. Dotted line indicated copy number variations annotated by the right vertical axis. Bev, bevacizumab; chemo, chemotherapy; VAF, variant allelic fraction

Serial CSF cfDNA sequencing was available in 8 patients at the time of baseline, disease progression during osimertinib 80 mg treatment, and the second progression on continuing osimertinib strategy, all of which had confirmed LM diagnosis (Fig. 5). For patient B266, the level of T790M decreased on the first progression of osimertinib 80 mg and decreased to an undetected level on the second progression of osimertinib 80 mg combined with bevacizumab. Multiple alterations including *EGFR*, *CDKN2A*, *BRCA2*, and *TP53* increased slightly on the first progression and decreased dramatically on the second progression (Fig. 5A). For patient B176, numerous alterations emerged at the time of osimertinib 80 mg resistance including *NKX2-1*, *AKT1*, *DLL3*, and *MCL* copy number gain as well as *EGFR*, *CTNNB1*, and *LRP1B* mutations, but dramatically decreased during second treatment with osimertinib 80 mg combined with chemotherapy except for *DLL3* (Fig. 5B). For patient B274, the allelic fraction of *T790M* and copy number of multiple genes including *AKT3*, *CD274*, *DDR2*, and *IL7R* decreased gradually during the first and second progression on osimertinib 80 mg. But copy number gain of 2 genes, *MCL1* and *NTRK1* were first identified from repeat CSF sample at the time of the second progression on 80 mg (Fig. 5C). Patient B222 received osimertinib 80 mg and then had the dose escalated to 160 mg. At the time of resistance to osimertinib 160 mg, copy number gain of *EGFR*, *NKX2-1*, *MCL1*, and *AKT3* were first detected (Fig. 5D). Another 4 patients with serial CSF were presented in Additional file 4: Fig. S2 indicating changes of multiple alterations possibly associated with osimertinib resistance in LM.

## Discussion

Given the increasing incidence of LM in patients with *EGFR*-mutated NSCLC, there has been a large unmet need to develop effective systemic therapies for LM. Previous studies mainly explored extracranially effective targeted drugs on LM which were based on genomic characterizations of extracranial tumors or plasma. In the current study, we presented CSF-private resistant mechanisms to osimertinib in patients with LM. Even though the included patients were heavily pre-treated and nearly 50% had ECOG PS score ≥2, 22 of them received genomically CSF-based targeted therapy and demonstrated a 72.7% neurological response and the observed radiological response was durable (3.2 months). For those who did not receive matched targeted therapy, continuing osimertinib 80 mg combined with other systemic or local therapy tended to achieve more clinical response. It also had significantly longer survival than a direct switch to an agnostic treatment in those with CNS-only progression. Serial CSF monitoring indicated tumor evolution of LM and that emerging alterations might be related to disease progression of LM.

LM was usually a late manifestation of advanced NSCLC with severe neurologic symptoms, and treatment option was limited by the time of diagnosis. In historical reports, a median OS of only 3.6 months was observed in heavily pre-treated patients with LM [14]. In this study, the majority of patients with LM reflected this specific population of multiply pre-treated status. Besides, treatments to target resistant mechanisms of like *EGFR C797S*, *MET* amplification, etc., were still under exploration [15, 16]. Following the novel algorithm of CSF-directed therapy in the first cohort, we reported improved clinical response and nearly double historical median OS: A duration of 3.2 months with intracranial response radiologically and a median OS of 7.2 months were seen in patients with CSF-directed targeted therapies. This benefit might be owing to overcoming resistant mutations detected in CSF. But still more investigation is warranted in this regard for a solid conclusion. In the second cohort who had osimertinib continuation as non-matched therapy, it also showed a longer OS of 7.8 months than the historical control. This benefit was not overestimated by selection bias in mutational profiles as 83.7% of patients harbored concurrent TP53/RB1/CDK4 which were previously reported to have a negative impact on targeted treatment. Further evaluation in prospective studies to confirm these is demanded.

The rationale of CSF-directed targeted therapies for LM was supported by previous studies. Paired analyses of the patient-matched primary tumor and BM has implicated that the activation of the *PI3K/AKT/mTOR* pathway and *cyclin-dependent kinase* (*CDK*) pathway may represent a site-specific contributor to BM development [17, 18]. Based on these results, a proof-of-concept study included patients with BM and *CDK* alteration who obtained therapeutic intracranial benefits from *CDK* inhibition [8]. Therefore, it seemed feasible to use BM-specific alterations to inform CNS-penetrant targeted therapy [19]. Though LM seemed biologically distinct from parenchymal BM, the strategy of site-specific treatment should be also highly suggested as leptomeninges is a special anatomic composition of the CNS [20]. But the effort was largely hindered by the inaccessibility of biopsy and surgical resection of LM, leading to the use of CSF-derived tumor components as a proxy to reveal the unique genetic profiles of LM [7, 11, 21, 22]. However, report on CSF-directed targeted therapy for patients with LM is still a paucity. In the current study, considering an increasing number of patients treated with osimertinib and the lack of effective CNS-penetrant treatments after progression, we carefully selected patients with EGFR-mutated NSCLC who progressed on osimertinib with LM as the progression site. CSF and paired tumor and plasma were collected for NGS if available. We found that more CSF-private alterations were detected as compared to the plasma. As a reference to previously reported resistant mechanisms to osimertinib, CSF had *C797S* mutation, *MET* amplification, cell cycle pathway alterations, etc., which were not detected in paired plasma, leading to 22 patients received matched targeted therapy as subsequent treatments. In addition to improved response and OS, there was also 1 long-term survivor (duration of response≥2 years) in this cohort. Taken together, our study presented evidence of choosing LM-specific targeted therapy based on molecular analysis of CSF.

Though CNS disease progression has been attributed to CNS-specific molecular alterations, in vitro drug diffusion properties and decreased ratio of CSF-serum drug concentration indicated that the existence of the blood-brain barrier (BBB) should also be blamed [19]. Two previous studies explored whether the strategy of increased drug delivery into CNS might be translated into more effective tumor kill in LM [23, 24]. One included patients with *EGFR*-mutated LM and proved that TKI rechallenge with dose escalation provided substantial activity after initial TKI failure. Most patients in this study were treated with the first- or second-line *EGFR*-TKIs [24]. Another study included osimertinib failure cases and had CNS progression as the primary indication to receive dose intensification strategy: dose escalation and combination with brain radiotherapy [23]. It showed a modest intracranial benefit. Another two cases also reported the promising efficacy of osimertinib in combination with bevacizumab or pemetrexed in LM [25, 26]. In our study, in addition to matched targeted therapy, non-matched therapies were adopted at progression. Continuing osimertinib with a combination of chemotherapy/bevacizumab/radiation had higher rate of clinical response than regimen switch. It also had a significantly longer survival than a direct switch to agnostic treatments in those with CNS-only progression, not in the systemic progression group. But what should be noticed was that based on our retrospective analysis with a small number of patients, it remained unclear whether the benefit of continuation and combination strategy should refer to additional treatments enhancing osimertinib efficacy in CNS control, or to their own intracranial efficacy. Both sides have some reasonable explanations. On the one hand, in those with CNS-only progression, we assumed that limited drug exposure of osimertinib in CNS might be one of the reasons for LM progression. Drug concentrations in CSF might increase by the destruction of BBB by other systemic treatments like chemotherapy and bevacizumab, and radiation [27]. For example, vascular endothelial growth factor (VEGF) was secreted by astrocytes, an important structural component of BBB for promoting endothelial cell barrier function. Bevacizumab, an anti-VEGF small molecule might therefore impair restriction from BBB and enhance drug diffusion into the CNS by combining with osimertinib [28, 29]. On the other hand, chemotherapy, bevacizumab, and brain radiation also had a modest efficacy on LM, respectively. In a word, our study provided some evidence of continuing osimertinib with additional therapy as an alternative when the standard of care was exhausted in LM. But suggestions based on this evidence should still be made with caution. Prospective studies in this regard are ongoing (NCT04148898, NCT04410796).

Serial cfDNA sequencing of CSF was available at baseline, first progression, and second progression on osimertinib in our study. Multiple alterations increased in allelic fraction or copy number at the time of first resistance to osimertinib but dramatically decreased during continuing osimertinib added with bevacizumab or chemotherapy. This phenomenon was consistent with the understanding of universal tumor killed by angiogenic or cytotoxic agents. For another patient who had osimertinb 2nd progression, several alterations emerged which were likely to be LM-specific resistance. A fourth patient received dose escalation to 160 mg after standard dose progression. During dose-escalation treatment, emerging genetic alterations were also detected which could not be overcome by osimertinib even with adequate drug delivery. These results indicated that serial monitoring of CSF might detect LM-specific resistant mechanisms, and some of which might be overcome by adding agnostic therapy. Studies with larger samples are needed to further explore this.

Previously, a proposed approach to the management of *EGFR*-mutant NSCLC progression on osimertinib was recommended: tumor biopsy of the progressive site should be accessed for evaluation of resistant mechanisms to indicate subsequent treatment. But for diffuse CNS involvement, a simple transition to CNS-penetrant systemic therapy was suggested [30]. Based on our results, we considered molecular analysis of CSF which was much more sensitive than the plasma in replace of tumor biopsy of LM to identify intracranial resistant mechanisms and indicate LM-specific treatment. Thus, we proposed a possible scheme as a complement in CNS progression. We expect that it will serve as a reference to future prospective study design on CSF-informed decision making for CNS metastases especially LM (Additional file 5: Fig. S3).

### Limitations

Firstly, this is a retrospective study with a small sample size. But it should be noticed that the incidence rate of CNS progression on osimertinib was relatively low (6% reported by the FLAURA trial), and the number of LM progression was assumed to be lower. Secondly, the superiority of matched therapy should be further reflected by prolonged survival than non-matched therapy among patients with the same resistant mechanisms. Limited sample size hampered this analysis. Besides, some patients in the matched cohort received agents for potentially druggable targets. These agents were also still under investigation. Thus, more prospective data in this regard is warranted.

## Conclusions

CSF might detect resistant mechanisms that led to matched targeted therapy in patients with LM. Besides, continuing osimertinib with an intensification strategy might achieve prolonged survival in patients with LM, especially those with CNS-only progression. While the intracranial response was observed, our conclusion was tempered by its retrospective nature and small cohort. A future step of exploration is needed.

## Supplementary information

**Additional file 1: Supplementary table 1.** Spreadsheet including matched regimens used in 22 patients with LM.

**Additional file 2: Fig. S1.** Non-matched therapy as post-osimertinib treatment for LM. A. Overall survival of non-matched therapy; B. Comparison of overall survival between osimertinib 80 mg combination (chemotherapy/bevacizumab/radiotherapy) and regimen switch in those with systemic progression.

**Additional file 3.** Spreadsheet including non-matched regimens used in 59 patients with LM.

**Additional file 4: Fig. S2.** Serial monitoring by CSF for osimertinib treatment in patients with LM. A. Patient B272: CSF cfDNA sequencing at baseline, disease progression during osimertinib 80 mg treatment and second progression on osimertinib 80 m combined with bevacizumab; B. Patient B273: CSF cfDNA sequencing at baseline, disease progression during osimertinib 80 mg treatment and second progression on osimertinib 80 m combined with chemotherapy; C. Patient B234: CSF cfDNA sequencing at baseline, disease progression during osimertinib 80 mg treatment and second progression on afatinib combined with bevacizumab; D. Patient B226: CSF cfDNA sequencing at disease progression during osimertinib 80 mg treatment and second progression on osimertinib 160 mg. Full line indicated mutations annotated by the left vertical axis; Dotted line indicated copy number variations annotated by the right vertical axis. Bev, bevacizumab; chemo, chemotherapy; VAF, variant allelic fraction.

**Additional file 5: Fig. S3.** A proposed schema of molecular analysis of CSF to inform site-specific targeted therapy for LM at osimertinib progression.

## Data Availability

The datasets used and/or analyzed in the current study were mostly presented in the main manuscript as well as additional supporting files; more details were available from the corresponding author and first authors on reasonable request.

## Abbreviations

LM: Leptomeningeal metastases
NSCLC: non-small cell lung cancer
CNS: Central nervous system
CSF: Cerebrospinal fluid
cfDNA: Cell-free DNA
TKIs: Tyrosine kinase inhibitors
RANO: Response Assessment in Neuro-Oncology
ctDNA: Circulating tumor DNA
NG: Next-generation sequencing
OS: Overall survival
ECOG-PS: Eastern Cooperative Oncology Group performance status
PR: Partial response
SD: Stable disease
CDK: Cyclin-dependent kinase
BBB: Blood-brain barrier
VEGF: Vascular endothelial growth factor
