# Supplementary figures and images for: Subsequent treatments beyond progression on osimertinib in EGFR-mutated NSCLC and leptomeningeal metastases
Source: BMC Med. 2022 May 30;20:197. doi: 10.1186/s12916-022-02387-0 (PMC9150343; doi:10.1186/s12916-022-02387-0)

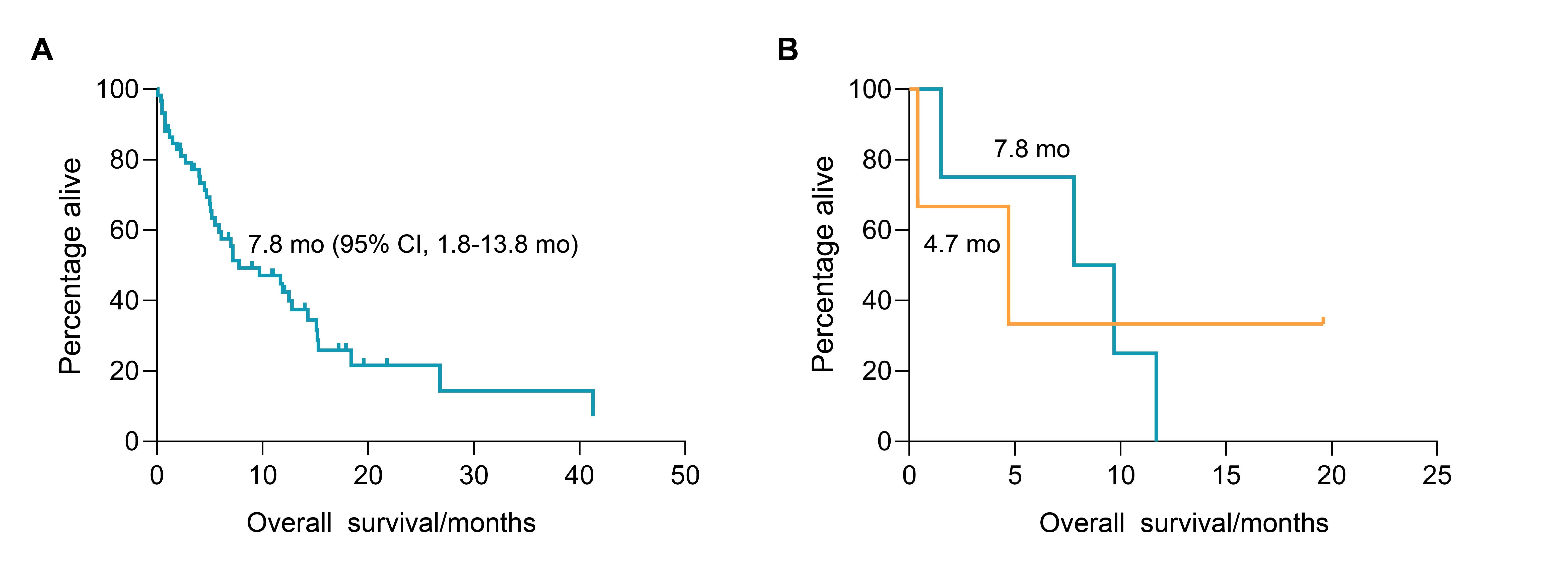

Supplement: Supplementary file 2 — Additional file 2: Fig. S1. Non-matched therapy as post-osimertinib treatment for LM. A. Overall survival of non-matched therapy; B. Comparison of overall survival between osimertinib 80 mg combination (chemotherapy/bevacizumab/radiotherapy) and regimen switch in those with systemic progression. [file 12916_2022_2387_MOESM2_ESM.jpg]

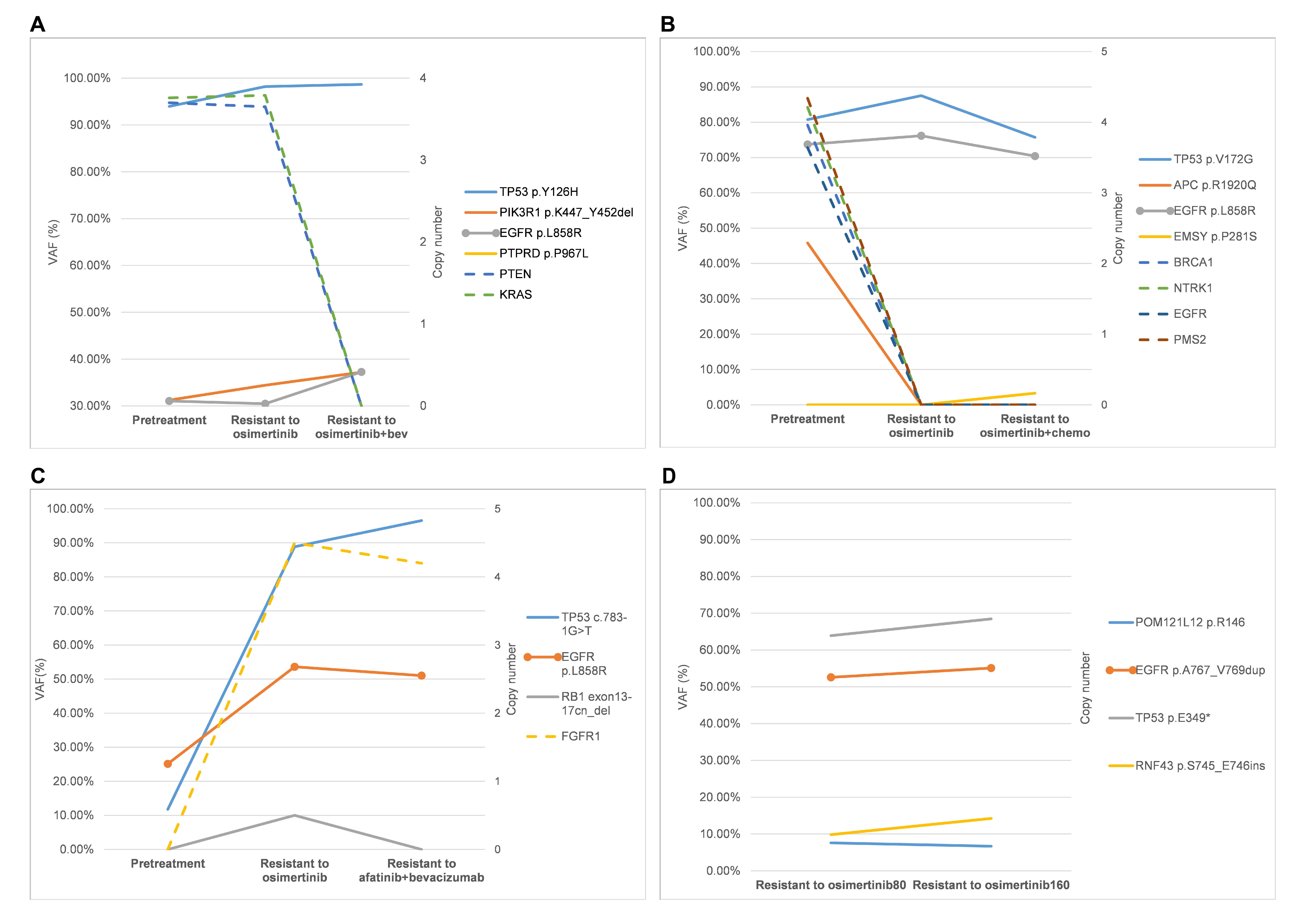

Supplement: Supplementary file 4 — Additional file 4: Fig. S2. Serial monitoring by CSF for osimertinib treatment in patients with LM. A. Patient B272: CSF cfDNA sequencing at baseline, disease progression during osimertinib 80 mg treatment and second progression on osimertinib 80 m combined with bevacizumab; B. Patient B273: CSF cfDNA sequencing at baseline, disease progression during osimertinib 80 mg treatment and second progression on osimertinib 80 m combined with chemotherapy; C. Patient B234: CSF cfDNA sequencing at baseline, disease progression during osimertinib 80 mg treatment and second progression on afatinib combined with bevacizumab; D. Patient B226: CSF cfDNA sequencing at disease progression during osimertinib 80 mg treatment and second progression on osimertinib 160 mg. Full line indicated mutations annotated by the left vertical axis; Dotted line indicated copy number variations annotated by the right vertical axis. Bev, bevacizumab; chemo, chemotherapy; VAF, variant allelic fraction. [file 12916_2022_2387_MOESM4_ESM.jpg]

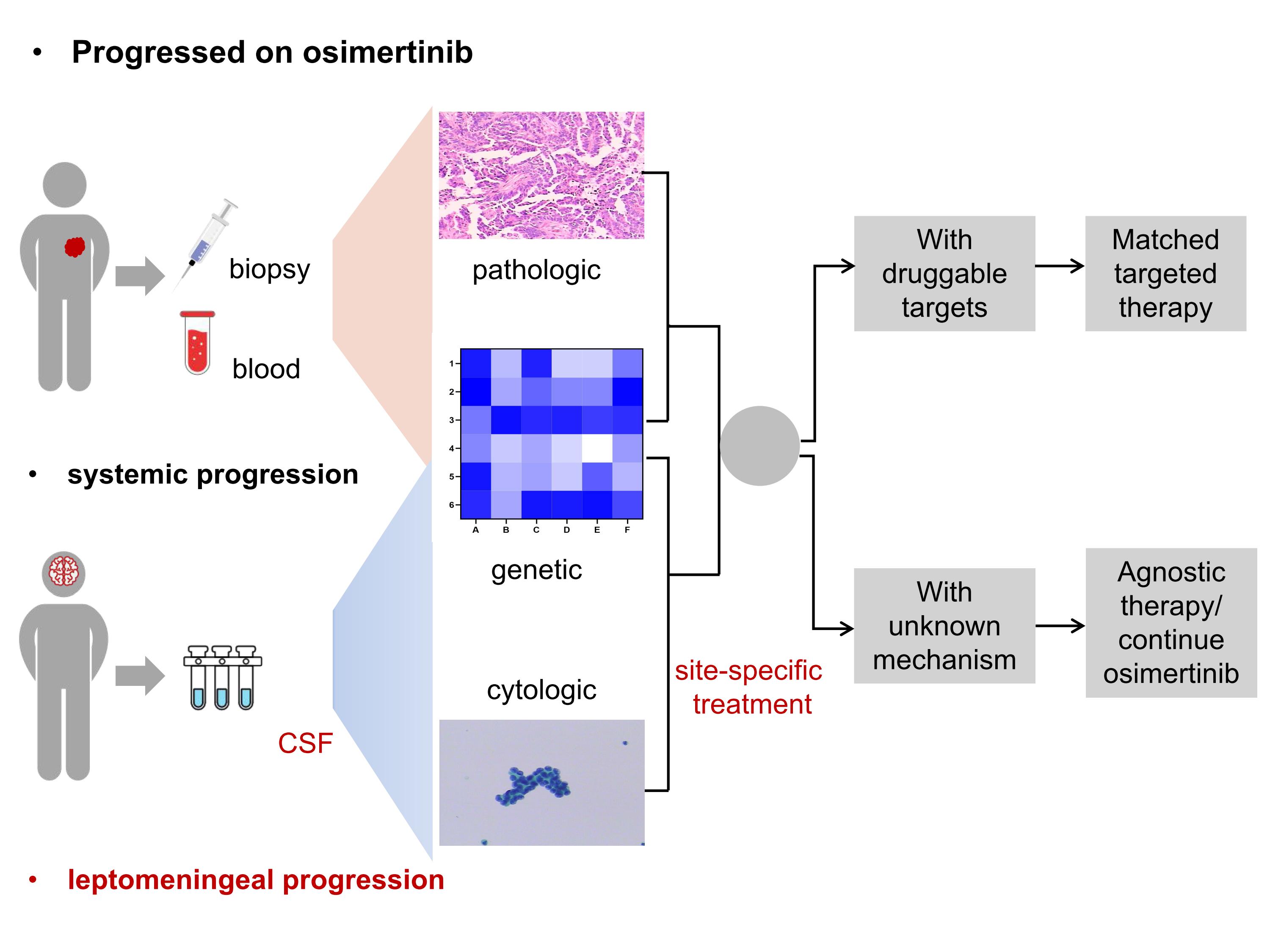

Supplement: Supplementary file 5 — Additional file 5: Fig. S3. A proposed schema of molecular analysis of CSF to inform site-specific targeted therapy for LM at osimertinib progression. [file 12916_2022_2387_MOESM5_ESM.jpg]
